# Fighting eimeriosis by using the anti-eimerial and anti-apoptotic properties of rhatany root extract

**DOI:** 10.3389/fimmu.2024.1430960

**Published:** 2024-07-11

**Authors:** Saleh Al-Quraishy, Rewaida Abdel-Gaber, Ghada Alamari, Andreas Meryk, Saeed El-Ashram, Esam M. Al-Shaebi, Mohamed A. Dkhil

**Affiliations:** ^1^ Department of Zoology, College of Science, King Saud University, Riyadh, Saudi Arabia; ^2^ Department of Pediatrics, Medical University of Innsbruck, Innsbruck, Austria; ^3^ College of Life Science and Engineering, Foshan University, Foshan, Guangdong, China; ^4^ Faculty of Science, Kafrelsheikh University, Kafr El-Sheikh, Egypt; ^5^ Department of Zoology and Entomology, Faculty of Science, Helwan University, Cairo, Egypt; ^6^ Applied Science Research Center, Applied Science Private University, Amman, Jordan

**Keywords:** Rhatany, *Eimeria papillata*, coccidiostats, apoptosis, goblet cells, mice

## Abstract

**Background:**

Over the last decade, extensive use of coccidiostats to treat and control *Eimeria* infection has developed drug resistance, prompting the search for new alternative therapies. Rhatany is proven to have various pharmacological properties.

**Objective:**

The present study aimed to *in vitro* and *in vivo* evaluate the effect of Rhatany roots extract (RRE) as an anti-eimerial and anti-apoptotic agent against murine eimeriosis induced by *Eimeria papillata*.

**Methods:**

Phytochemical screening by gas chromatography-mass spectrometry analysis (GC-MS) was used to detect active compounds in RRE. *In vitro* anti-eimerial activity of RRE (200, 100, 50 mg/ml), amprolium, phenol, Dettol™, and formalin were studied after incubation with non-sporulated *Eimeria* oocysts. For the *in vivo* study, twenty-five male C57BL/6 mice were randomly allocated into five groups. Animals in the first group were just given distilled H_2_O, while those in the second group were given 200 mg/kg RRE for 5 days. The *Eimeria* parasite’s oocysts were infected into the third, fourth, and fifth groups. For treatment, RRE (200 mg/kg) and amprolium (120 mg/kg) were orally given to the 4^th^ and 5^th^ groups for five days, respectively. All mice were euthanized, on day 5 post-infection, to collect the jejunal tissues under study. Investigations were undertaken into the oocyst output in feces and goblet cells in mice jejuna. Assays for glutathione peroxidase (GPx), hydrogen peroxide (H_2_O_2_), and myeloperoxidase (MPO) were also performed. In jejunal tissue, cysteine aspartic acid protease-3 (Caspase-3) was counted using immunohistochemistry, while BCL2-associated X protein (Bax) and B-cell lymphoma-2 (BCL-2) were assayed using ELISA. In addition, mRNA expression of the goblet cell response gene (MUC2) was detected using real-time PCR.

**Results:**

Phytochemical screening by GC-MS demonstrated the presence of 22 compounds in the RRE. The *in vitro* study revealed that RRE significantly inhabited the oocyst sporulation in a dose-dependent manner. By day 5 after infection with the *Eimeria* parasite, the number of oocysts in mice feces was significantly reduced after RRE treatment (1.308 × 10^6^ ± 1.36 × 10^5^ oocysts/g feces) compared to the infected group (5.387 × 10^6^ ± 4.29 × 10^5^ oocysts/g feces). Moreover, the *Eimeria* infection reduced the number of goblet cells of mice jejuna and its specific gene, MUC2. The treatment with RRE increased the number of goblet cells/villus from 3.45 ± 0.17 to 6.04 ± 0.23, associated with upregulation for MUC2 from 0.26 to 2.39-fold. Also, the *Eimeria* experimental infection lowered the activity of the antioxidant enzyme represented by GPx (23.99 ± 3.68 mg/g tissue), while increasing the stress parameters of hydrogen peroxide (0.07 ± 0.01 mM/g) as well as the activity of MPO (66.30 ± 3.74 U/mg). The production of apoptotic markers including Caspase-3 (68.89 ± 2.67 U/g) and Bax (159.05 ± 6.50 pg/ml) was significantly elevated while decreasing the anti-apoptotic marker of BCL2 (0.42 ± 0.07 pg/ml). Our study proved that RRE significantly reduced oxidative stress, and apoptotic markers as well as the inflammatory activity of MPO. Also, antioxidant enzyme and anti-apoptotic activity in the jejunum of *E. papillata*-infected mice were enhanced after RRE treatment.

**Conclusion:**

Our study highlights the potential of RRE as a natural solution for coccidiosis management by modulating apoptosis in *E. papillata* host cells. However, further research is needed to fully understand the underlying mechanisms and enhance our understanding of its therapeutic efficacy.

## Introduction

Coccidiosis continues as one of the most destructive protozoan diseases of wild and domestic animals worldwide and causing economic losses (1). The causative agent for this disease is the host-specific apicomplexan species belonging to the genus *Eimeria* (Eucoccidiorida, Eimeriidae) which is transmitted via the fecal-oral route of oocysts, which release infectious sporozoites in the intestine (2). *Eimeria papillata* represents a convenient model for studying animal coccidiosis through its intracellular development within the predilection site in the mouse jejunum (3). The mucus layer coating the gastrointestinal tract is considered the front line of innate host defense, largely because of the secretory products of intestinal goblet cells, including the mucin MUC2 (4). Indeed, *Eimeria* infection has been associated with an increase in the incidence of pathological conditions, inflammation, and oxidative stress that affects the general body performance (1).

Coccidiosis can be treated with a range of medications, but usage of these medications contributed to multidrug resistance and an overabundance of parasite infection in tissues. Inappropriately, no new drugs have been licensed for usage, which has heightened the desire for innovative anti-eimerial options derived from natural origin (5). Among, other available options, different compounds obtained from botanicals have shown excellent and admirable antieimerial and other therapeutic effects (6). One advantage of employing natural extracts is that they reduce the possibility of developing resistance; also, the residues of such extracts natural items in animal products are safe for human consumption and have no negative health consequences (7). In Saudi Arabia, several medicinal plant extracts were proven for their anti-eimerial activity such as *Ziziphus spina*-*christi* (8), *Glycyrrhiza glabra* (9), *Salvadora persica* (6), *Salvadora persica* (10), *Astragalus membranaceus* (11), and *Azadirachta indica* (12).

Rhatany, also known by its Latin name *Krameria lappacea*, boasts a rich history of traditional medicinal use and diverse applications across industries, captivating researchers and enthusiasts alike (13). The roots of this plant are utilized as a traditional herbal remedy. Phytochemical analysis of rhatany has revealed a variety of bioactive compounds including phenolics, flavonoids, tannins, lignan derivatives, oligomeric proanthocyanidins, and benzofuran derivatives (14). The medicinal properties attributed to rhatany include antioxidant (15), anti-inflammatory (16), antidiabetic (17), anticancer (18), and antimicrobial (19) activities. Additionally, its constituents are applied in alternative and modern medicine, for example, for the treatment of diverse illnesses, among these, infections of the respiratory airways and gastrointestinal disorders (20).

Our objective was to evaluate the impact of Rhatany roots extract (RRE) in mitigating oxidative stress induced by *Eimeria papillata*. Additionally, we sought to investigate the response of goblet cells in regulating gene expression and apoptotic markers both during infection and following the administration of the extract.

## Materials and methods

### Plant collection and extract preparation

Rhatany roots were obtained from the local market in Riyadh, Saudi Arabia. A taxonomist at the Herbarium of the College of Science (King Saud University), validated the plant’s botanical identity. Roots were crushed in an electric blender to obtain a coarse powder. The 70% rhatany root methanolic extract (RRE) was prepared using the process cited in Alamari et al. (21).

### Gas chromatography-mass spectrometry analysis

The methanolic rhatany extract was subjected to GC-MS analysis using a Thermo Scientific ™ Trace GC Ultra and ISQ™ Single Quadruple MS (Thermo Fisher Scientific, Waltham, MA, USA) (22). Identification of the mass spectrum was conducted regarding the Wiley/NBS mass spectral library.

### Protozoan parasite preparation

To prepare the oocysts for model mice infection, *Eimeria papillata* were collected and passaged in laboratory mice (*Mus musculus*). The portion of the produced oocysts in faeces was kept being used in the *in vitro* study and the other portion was incubated in 2.5% potassium dichromate (K_2_Cr_2_O_7_) for sporulation and then used in the *in vivo* experiment.

### *In vitro* oocyst sporulation

The *in vitro* oocyst sporulation was carried out in small Petri dishes. The first group was filled with distilled water, serving as a negative control. The second group contained K_2_Cr_2_O_7_ (2.5%) and served as a positive control. Successive groups received escalating doses of RRE (200, 100, and 50 mg/ml, respectively). Additionally, one group contained amprolium (8.3 mg/ml), another was filled with Dettol™ (109 µl), followed by one with phenol (25 µl), and another with formalin (5%). Each petri dish contained 1×10^5^ unsporulated *E. papillata* oocysts, which were incubated at 25°C for 72 and 96 hr. Sporulation of the oocysts was monitored by examining sporocysts using an Olympus compound microscope (Olympus Co., Tokyo, Japan). Sporulation and inhibition (%) were calculated according to Thagfan et al. (23).

### *In vivo* infection and experimental design

Twenty-five male C57BL/6 mice were used (aged 9–10 weeks and weighted 20–25 g). Mice were used in the experiment following the institution’s guidelines on the care and use of animals in research (approval no. KSU-SE-23–127). Animals were kept at standard laboratory conditions and allowed food and water *ad libitum*. The animals were divided into 5 groups, each comprising five mice. The first group served as the infection-free control group and was administered distilled water. In contrast, the second group received daily oral gavage inoculations of 200 mg/kg RRE (24). The third was considered as an infected group with 10^3^ sporulated oocysts of *E. papillata* (11). The fourth and fifth groups were considered as infected-treated groups as they orally infected with 10^3^ sporulated oocysts of *E. papillata* (11). Following infection, the fourth group was treated with 200 mg/kg RRE for 5 consecutive days, while the fifth group received a 5-day treatment with 120 mg/kg Amprolium (25). On the 5th day post-infection (p.i.), oocyst shedding was quantified in fecal pellets using a McMaster chamber and expressed as the number of Eimeria oocysts per gram of wet feces. Following that, all mice were euthanized, and portions of the jejunum were preserved for the following: (a) formalin buffered phosphate (10%) was utilized for histological and immunohistochemical analysis. (b) In small tubes maintained at -80°C to investigate the oxidative status and protein expression (c) RNA later® (Invitrogen, Carlsbad, CA) was utilized for molecular analysis and kept at -80°C.

### Goblet cell response

Tissue paraffin sections for identification of goblet cells in jejunum were prepared according to Adam and Caihak (26). Sections were stained with Alcian blue and countered with eosin (27). Jejunal sections were examined and photographed by an Olympus B×61 microscope (Tokyo, Japan). For each mouse, the number of goblet cells in the jejunum was counted in at least ten well-oriented villous crypt units (VCUs) and the results were presented as the mean number.

### Immunohistochemical staining of caspase-3

Detection of cysteine aspartic acid protease-3 (Caspase-3) was performed according to Dkhil et al. (28). Jejunal sections (5 µm) were deparaffinized and treated with 3% H_2_O_2_ for 10 min. Jejunal sections were incubated at 4°C with 1:100 dilution of mouse anti-caspase-3 antibodies (Santa Cruz Biotechnology, CA, USA) in phosphate-buffered saline (PBS). Following removal of the primary antibodies and repetitive rinsing in PBS, sections were incubated with a 1:2.000 dilution of biotin-conjugated secondary antibody (Santa Cruz Biotechnology). Sections were counterstained with hematoxylin (Sigma Chemical Co.) and re-incubated for 15 min with streptavidin which was labeled with horseradish peroxidase. All sections were photographed using an Olympus B×61 microscope (Tokyo, Japan). For each mouse, the number of positive cells in the jejunum was counted in at least ten well-oriented villous crypt units (VCUs) and the results were presented as the mean number.

### Biochemical analysis

Pieces of jejunum were weighed and then homogenized in an ice-cold medium with 50 mM Tris-HCl and 300 mM sucrose. After centrifugation (10 min at 500×g at 4°C), the supernatant was used for biochemical assays. Using the method of Paglia and Valentine (29), the concentration of glutathione peroxidase (GPx) was calculated in the jejunum homogenate. For hydrogen peroxide (H_2_O_2_), Aebi (30) method was performed. Absorbance was measured with Spectra MAX 190 supported by the software SoftMax^®^ Pro v.6.3.1.

### Myeloperoxidase activity

The activity of myeloperoxidase (MPO) was measured in the jejunum homogenate following the method of Bradley et al. (31). One unit of enzyme activity was defined as the amount of MPO that caused a change in absorbance measured at 460 nm for 3 min. Absorbance was measured with Spectra MAX 190 supported by the software SoftMax^®^ Pro v.6.3.1. MPO activity was expressed as U/mg tissue.

### Quantitative real-time polymerase chain reaction

Total RNA was isolated from the jejunum using Trizol (Invitrogen). RNA samples were transformed to cDNA using reverse transcription (Qiagen, Hilden, Germany). The quantitative real-time PCR (qRT-PCR) was performed using the ABI Prism^®^ 7500HT sequence detection system (Applied Biosystems, Darmstadt, Germany) with SYBR^®^ green PCR master mix (Qiagen, Hilden, Germany). Goblet cell response gene (MUC2) mRNA expression was determined using Qiagen qRT-PCR primers (Mm_Muc2_2_SG, Cat. No. Mm_Muc2_2_SG). The levels of mRNA had been normalized to glyceraldehyde-3-phosphate dehydrogenase (GAPDH) (Mm_Gapdh_3_SG, Cat. No. QT01658692). The Ct method (2^−ΔΔCT^) was described by Livak and Schmittgen (32) to evaluate the fold change in mRNA expression.

### Sandwich enzyme-linked immunosorbent assay

Using mouse ELISA kits (MyBioSource, USA), the level of apoptotic markers including BCL2-associated X protein (Bax) and B-cell lymphoma-2 (BCL-2) were investigated following the protocol instructions. Caspase-3 was assessed using a colorimetric test kit (Sigma-Aldrich, USA). Optical densities (OD) of outcomes from the jejunal samples were measured using the Bio-Rad IMark Microplate Reader SW 1.04.02.E. Based on a standard curve, OD values were converted to concentrations and presented as U/g (for caspase-3) and pg/ml (for Bax and BCL2).

### Statistical analysis

One-way ANOVA in SigmaPlot® version 11.0 (Systat Software, Inc., Chicago, IL, USA) was used to analyze data. Group-wise comparisons were performed using Duncan’s test. Values were presented as mean ± standard deviation (SD). *p*-value ≤ 0.05 was indicated statistical significance.

## Results

The results of GC-MS for RRE at different peak areas and retention times are presented in **Supplementary Figure S1** and **Table 1** showing 22 active phytochemical components. The major compounds identified in RRE were 1,2-benzenedicarboxylic acid, bis(2-methylpropyl) ester; hexadecanoic acid, methyl ester; 9,12-octadecadienoyl chloride; oleic acid; 9,12-octadecadienoic acid; arnebin 7; 2-(pentamethylbenzoyl) thiophene; estr-4-en-3-one; prasterone; diisooctyl phthalate; methyl abietate; γ-sitosterol; stigmasta-3,5-diene; and β-sitosterol (**Table 1**).

**Table 1:** Identification of phytochemical compounds by GC-Mass in RRE.

RT (min)	Proposed compound	MW	Peak area %	Formula
7.50	4H-Pyran-4-one, 2,3-dihydro-3,5-dihydroxy-6-methyl-	144	0.69	C_6_H_8_O_4_
7.60	N-Formyl-valine	145	0.73	C_6_H_11_NO_3_
8.91	5-Hydroxymethylfurfural	126	18.54	C_6_H_6_O_3_
11.14	1-Tetradecene	196	0.34	C_14_H_28_
11.52	4-Methylphthalaldehyde	148	0.64	C_9_H_8_O_2_
12.80	2,4-Di-tert-butylphenol	206	0.58	C_14_H_22_O
12.89	2-Acetylhydroquinone	152	0.90	C_8_H_8_O_3_
13.75	2-Dodecanol	186	0.46	C_12_H_26_O
16.95	1,2-Benzenedicarboxylic acid, bis(2-methylpropyl) ester	278	1.74	C_16_H_22_O_4_
17.51	Hexadecanoic acid, methyl ester	270	1.87	C_17_H_34_O_2_
19.21	9,12-Octadecadienoyl chloride	298	1.65	C_18_H_31_ClO
19.28	Oleic Acid	282	5.80	C_18_H_34_O_2_
19.60	9,12-Octadecadienoic acid	280	1.65	C_18_H_32_O_2_
20.19	Arnebin 7	272	20.29	C_16_H_16_O_4_
20.35	2-(Pentamethylbenzoyl)thiophene	258	12.87	C_16_H_18_OS
21.02	Estr-4-en-3-one	258	3.27	C_18_H_26_O
22.70	Prasterone	288	1.43	C_19_H_28_O_2_
23.24	Diisooctyl phthalate	390	4.67	C_24_H_38_O_4_
23.92	Methyl abietate	316	3.92	C_21_H_32_O_2_
25.27	γ-Sitosterol	414	13.88	C_29_H_50_O
28.07	Stigmasta-3,5-diene	396	1.31	C_29_H_48_
31.40	β-Sitosterol	414	2.77	C_29_H_50_O

**Figure 1:**
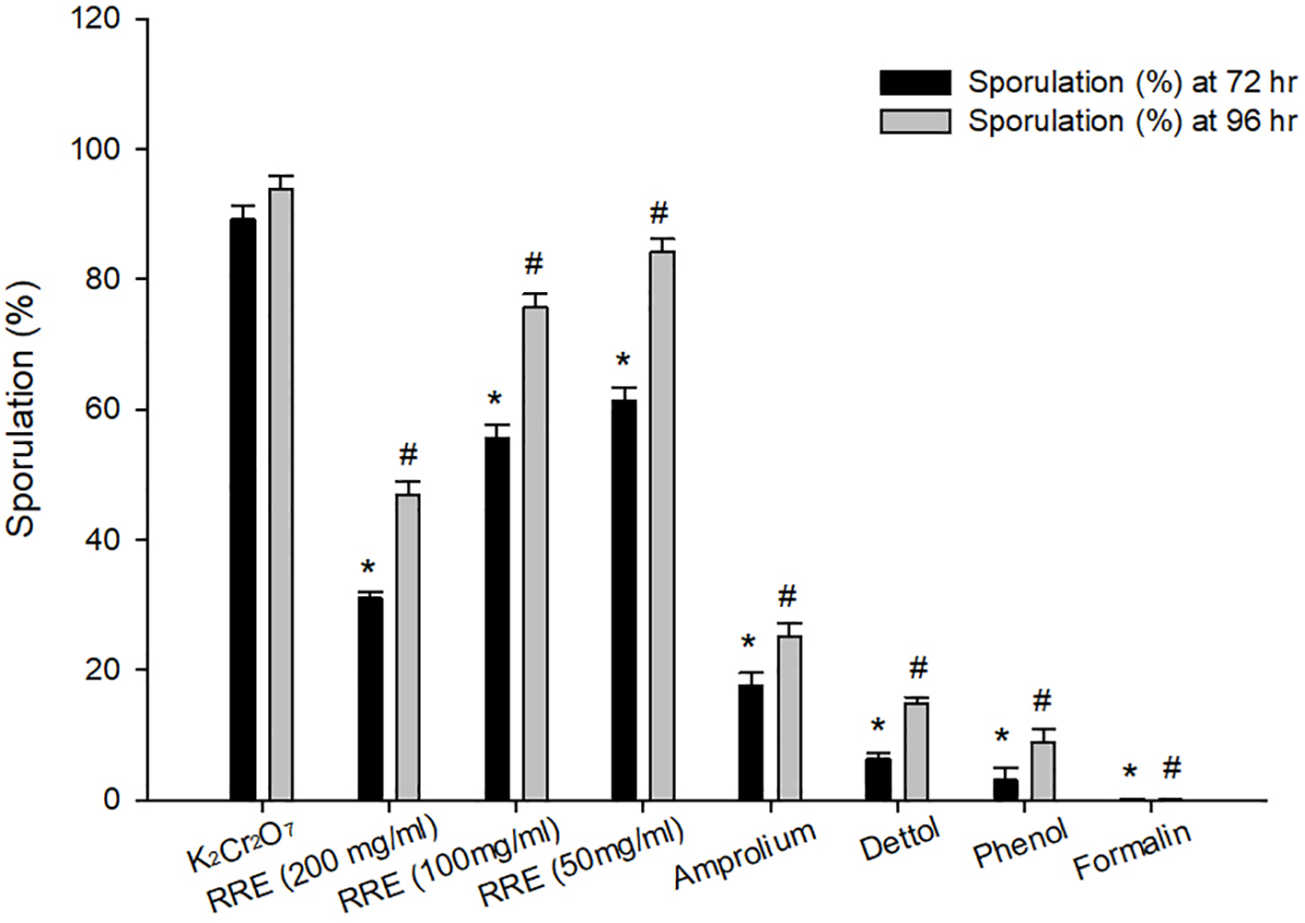
Sporulation percentage at 72 and 96 hrs for different treatments. ^*^ Significance change (p ≤ 0.05) at 72 hr concerning those treated with K_2_Cr_2_O_7_, ^#^ Significance change (p ≤ 0.05) at 96 hr concerning those treated with K_2_Cr_2_O_7_.

**Figure 2:**
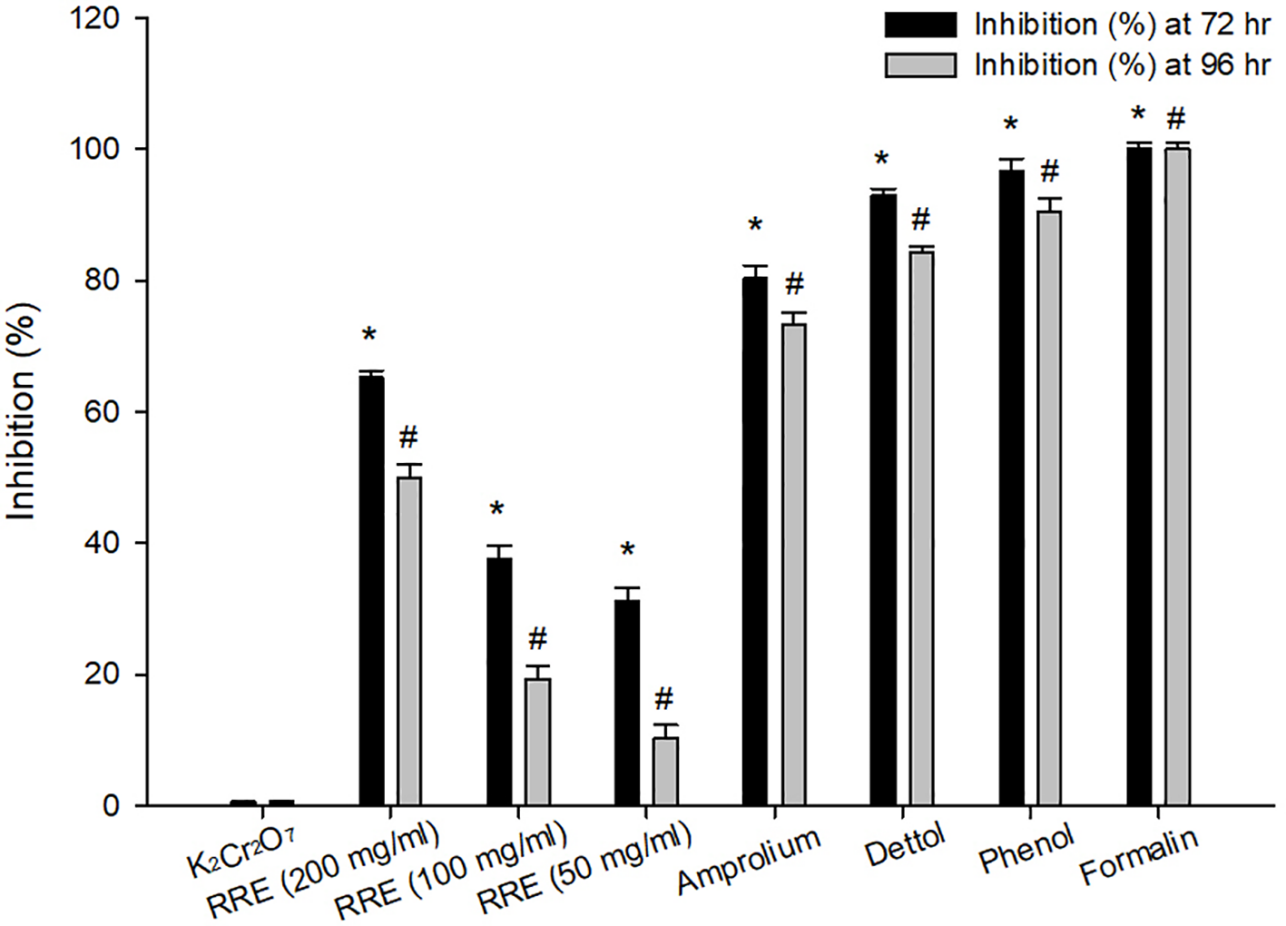
Inhibition percentage at 72 and 96 hrs for different treatments. ^*^ Significance change (p ≤ 0.05) at 72 hr concerning those treated with K_2_Cr_2_O_7_, ^#^ Significance change (p ≤ 0.05) at 96 hr concerning those treated with K_2_Cr_2_O_7_.

At 72 and 96 hrs, oocyst incubation with K_2_Cr_2_O_7_ (2.5%), RRE (200, 100, and 50 mg/ml), amprolium, phenol, and Dettol™ showed different levels of sporulation (**Figure 1**). After incubation with formalin, the unsporulated *E. papillata* oocysts showed no rate of sporulation. Incubation with RRE (200 mg/ml) for 72 to 96 hrs inhibited oocyst sporulation by 65.23% and 50.01%, respectively (**Figure 2**). RRE (100 and 50 mg/ml), amprolium, Dettol™, and phenol induced variable inhibition levels at 96 hr of 19.33%, 10.33%, 73.18%, 84.16%, and 90.47%, respectively.

**Figure 3:**
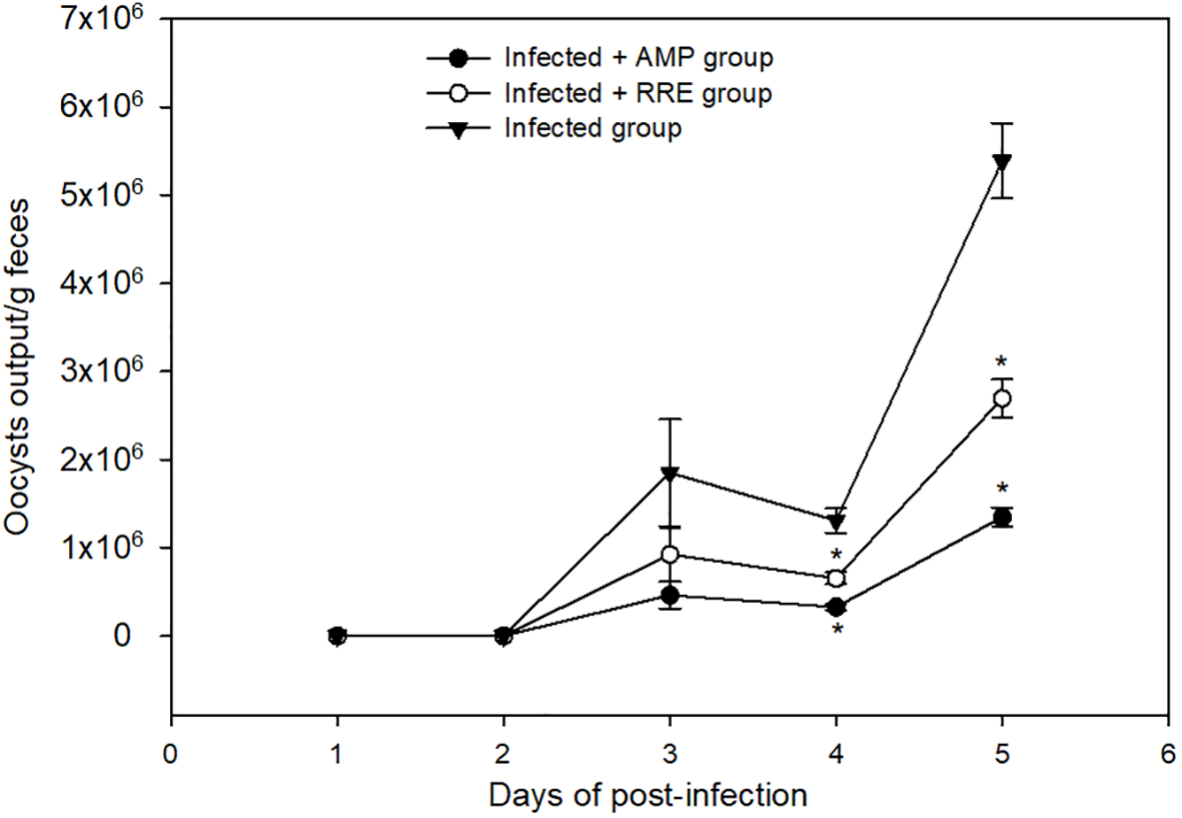
Oocyst output in mice infected with *Eimeria papillata* and for infected-treated groups with 200 mg/kg RRE and 120 mg/kg AMP. * significance (p ≤ 0.05) between infected and treated groups.

The shedding of the *Eimeria* oocysts started from three days of infection and reached its highest peak on the 5^th^ day p.i. RRE decreased the number of oocysts released in feces from 5.387 × 10^6^ ± 4.29 × 10^5^ to 1.308 × 10^6^ ± 1.36 × 10^5^ oocysts/g feces (**Figure 3**). RRE had a maximum anti-eimerial effect as it gave the best efficacy superior to amprolium (1.850 × 10^6^ ± 6.04 × 10^5^ oocysts/g feces) (**Figure 3**).

**Figure 4:**
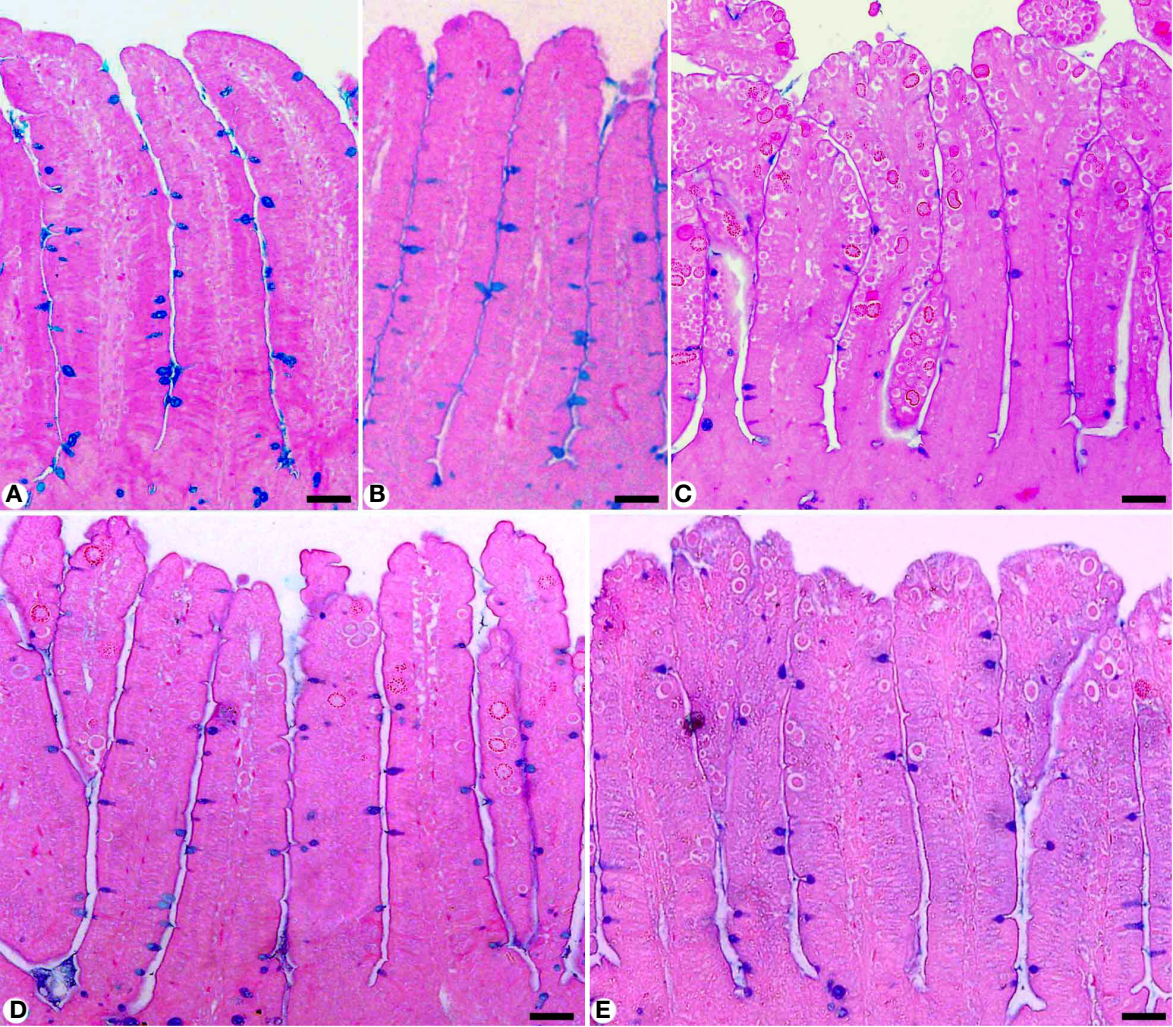
Jejunal sections stained with alcian blue and countered with eosin to identify the goblet cells in **(A)** control group, **(B)** non-infected-treated group with 200 mg/kg RRE, **(C)** infected group with *E*. *papillata*, **(D)** infected-treated group with 200 mg/kg RRE, and **(E)** infected-treated group with 120 mg/kg AMP. Goblet cells were counted in 10 well-oriented villus-crypt units (VCU). Scale bar = 100µm.

**Figure 5:**
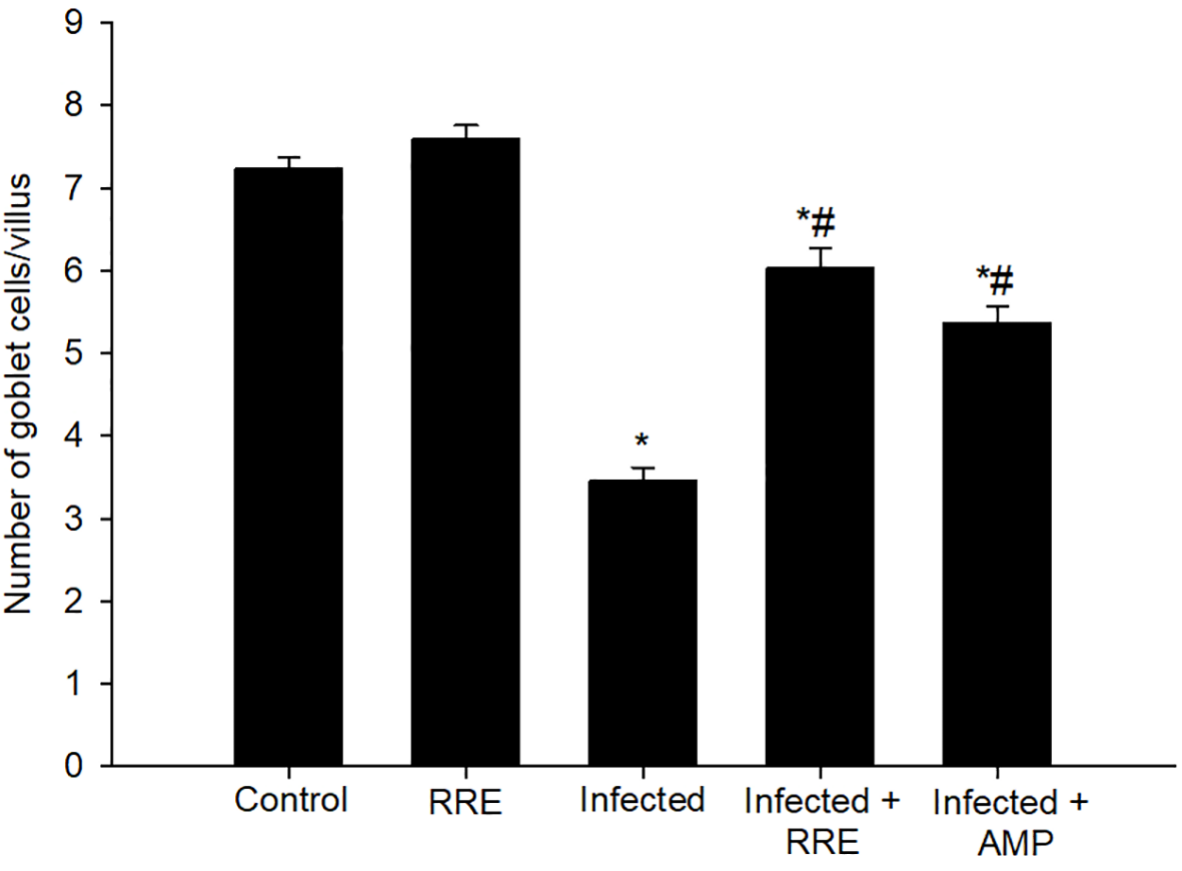
Changes in the number of jejunal goblet cells in villi of the control group, treated group with 200 mg/kg RRE, infected group with *E. papillata*, and infected-treated groups with 200 mg/kg RRE and 120 mg/kg AMP. Values are means ± SD. ^*^ significant change (p ≤ 0.05) concerning the control group, ^#^ significance change (p ≤ 0.05) concerning the infected group with *E. papillata*.

**Figure 6:**
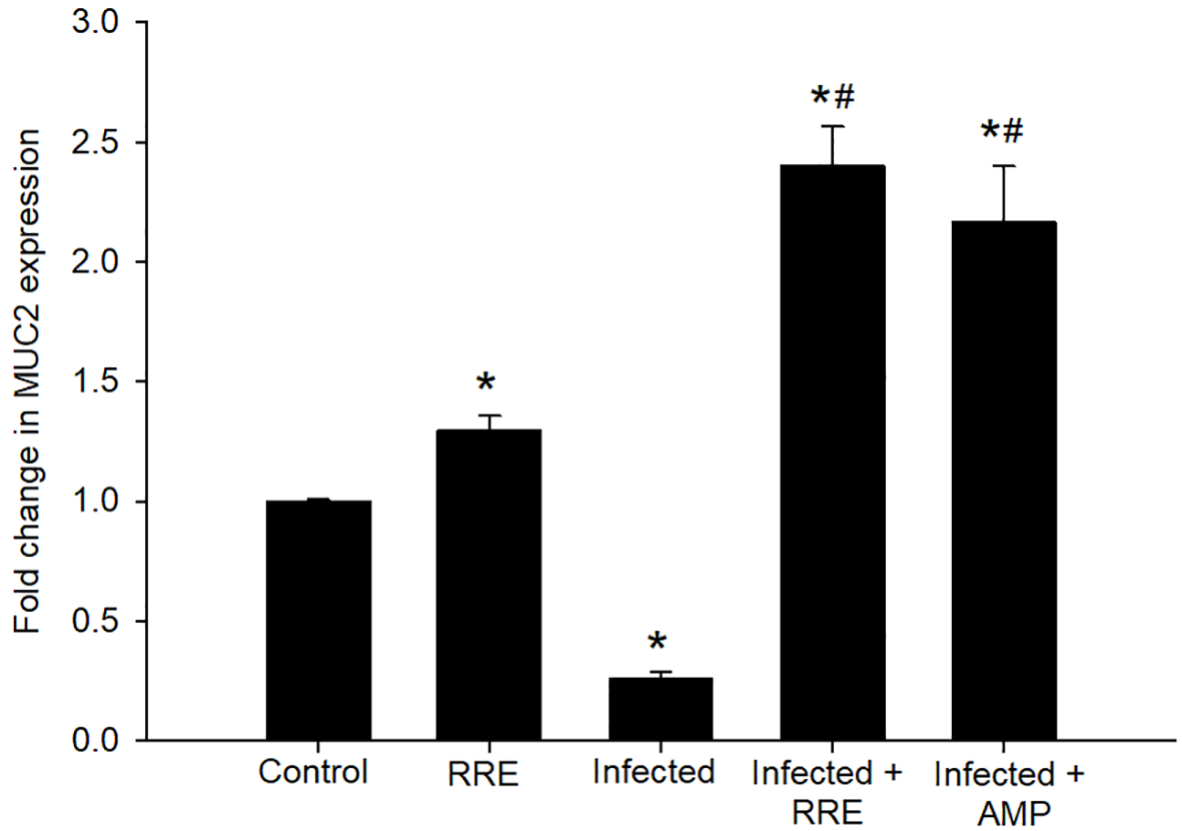
mRNA expression of *MUC2* in the jejunal samples from the different experimental groups. The expression values obtained by RT-PCR analysis were normalized to the reference gene GAPDH mRNA level and are shown as fold induction (in log 2 scale) relative to the mRNA level in the control. ^*^ significant change (p ≤ 0.05) concerning the control group, ^#^ significance change (p ≤ 0.05) concerning the infected group with *E. papillata*.

**Figure 7:**
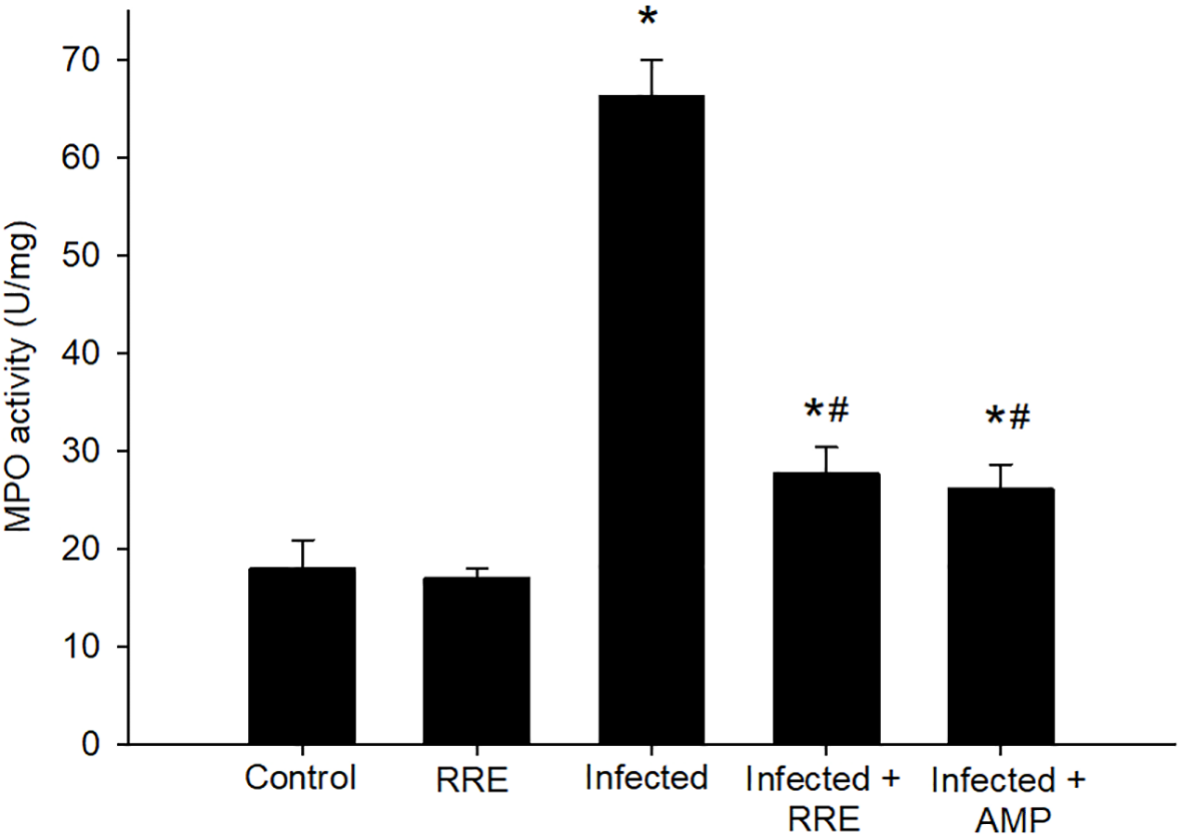
MPO activity in the jejunal samples from the different experimental groups. ^*^ significant change (p ≤ 0.05) concerning the control group, ^#^ significance change (p ≤ 0.05) concerning the infected group with *E. papillata*.

In Alcian blue stained sections of the mice jejunum (**Figure 4**), the number of goblet cells reduced significantly after *Eimeria* infection (3.45 ± 0.17/VCU) but increased after RRE treatment to 6.04 ± 0.23/VCU more than those treated with amprolium (5.37 ± 0.20/VCU) (**Figure 5**). Similarly, a significant downregulation in the mRNA expression of the *MUC2* gene that was secreted from goblet cells in the mice jejunum due to *Eimeria* infection (**Figure 6**). Upon treatment, RRE was able to significantly upregulate the *MUC2* gene expression from 0.26 to 2.39-fold associated with the number of goblet cells in the intestinal villi. Moreover, MPO which is considered a marker for neutrophil infiltration into the jejunal tissue was significantly increased during the *Eimeria* infection (66.30 ± 3.74 U/mg) and decreased after RRE treatment (27.71 ± 2.64 U/mg) (**Figure 7**).

**Figure 8:**
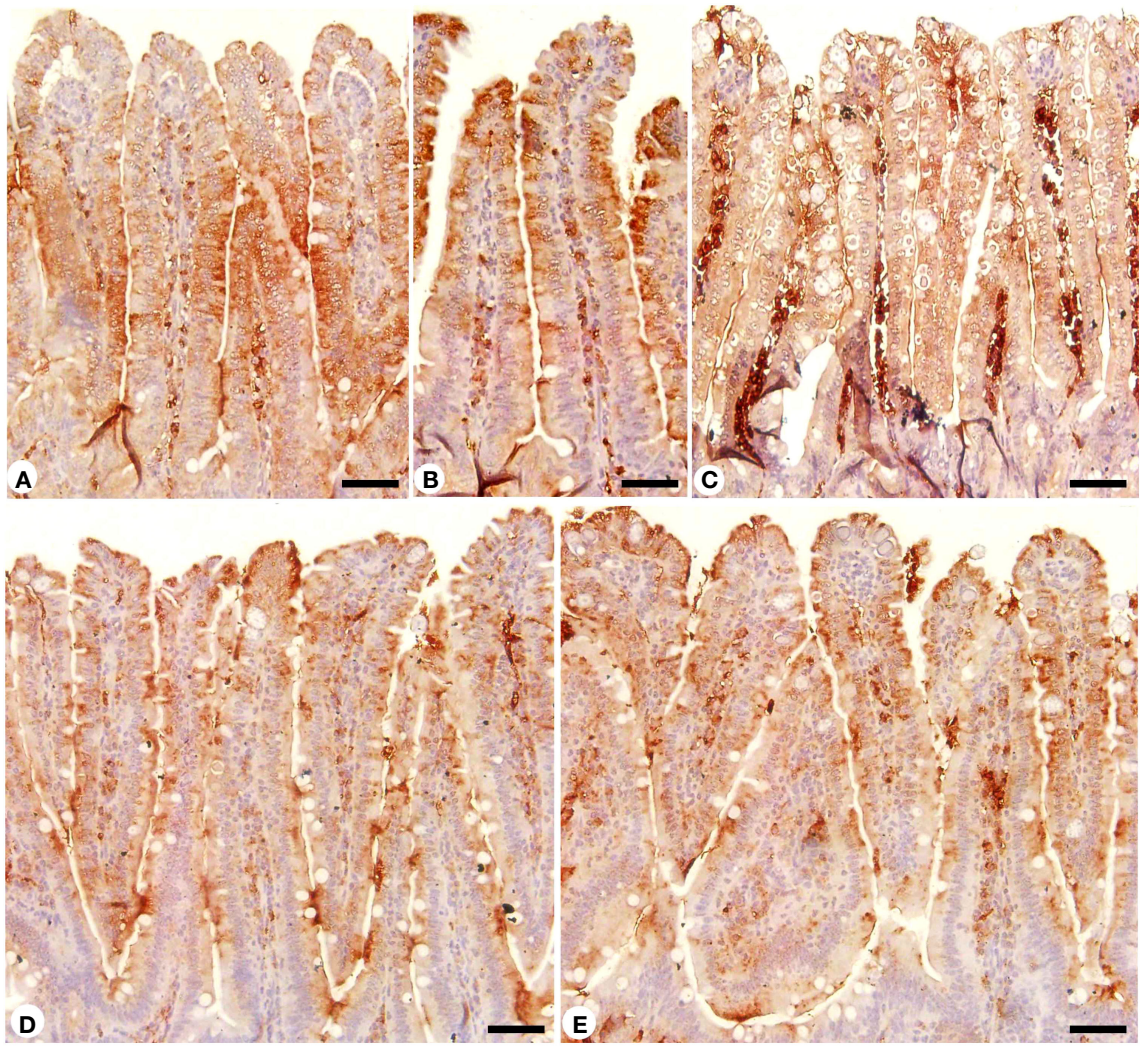
Immunohistochemical localization of caspase-3 in the jejuna of the different experimental groups. **(A)** control non-infected jejunum. **(B)** non-infected-treated group with 200 mg/kg RRE. **(C)** *E*. *papillata* infected jejunum with an increased number of caspase-3 positive cells. **(D, E)** infected-treated mice groups (200 mg/kg RRE and 120 mg/kg AMP, respectively) with a decreased number of caspase-3 positive cells. Scale bar = 50µm.

**Figure 9:**
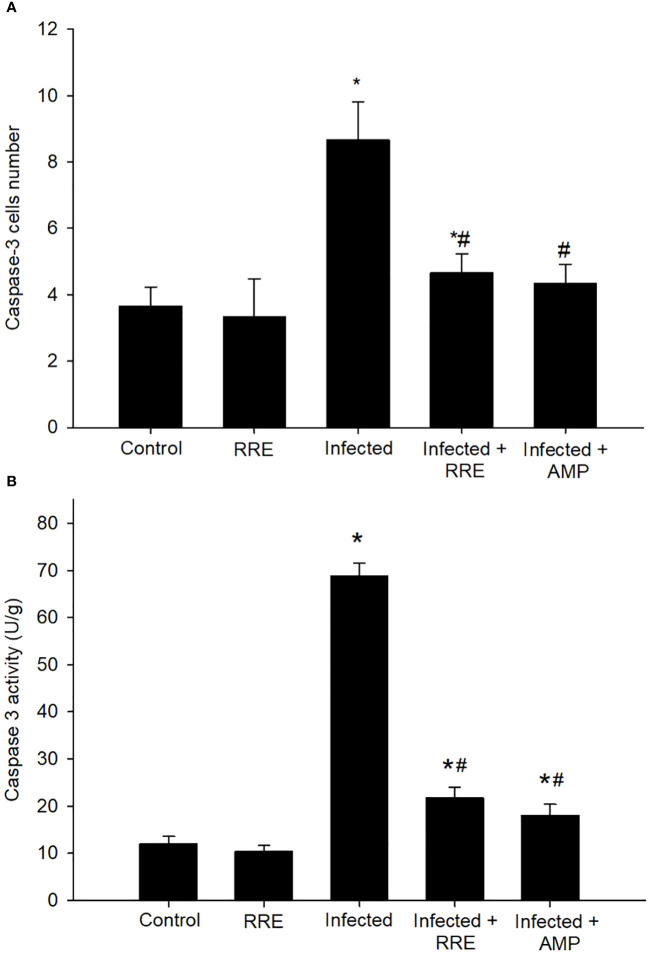
Caspase-3 in the jejunal samples from the different experimental groups. **(A)** Positive caspase-3 cell number. **(B)** Caspase-3 level. ^*^ significant change (p ≤ 0.05) concerning the control group, ^#^ significance change (p ≤ 0.05) concerning the infected group with *E*. *papillata*.

Jejunal sections from different experimental groups were stained for immunohistochemical investigation of caspase-3 expression which is considered a key player for apoptosis (**Figures 8**, **9**). It showed that the *Eimeria* infection induced an elevation of the caspase-3 expression level which was encoded by the caspase-3 gene with a number of positive cells reaching 8.66 ± 1.15 in the infected group compared to the normal status in the control group 3.66 ± 0.57 (**Figure 9**). This elevation may activate the cell death mechanisms in the infected group by intrinsic apoptotic genes. Upon treatment, caspase-3 expression significantly changed to decrease the immunoreactivity in the jejuna of mice in contrast to the infected group to be 4.66 ± 0.57 in the infected-treated RRE group and 4.33 ± 0.57 in the infected-treated AMP group. Similarly, a significant upregulation in the level of caspase-3 that was secreted in the mice jejunum due to *Eimeria* infection (**Figure 9**). Upon treatment, RRE was able to significantly downregulate the caspase-3 level from 68.89 ± 2.67 U/g to 21.72 ± 2.30 U/g in the intestinal villi.

**Figure 10:**
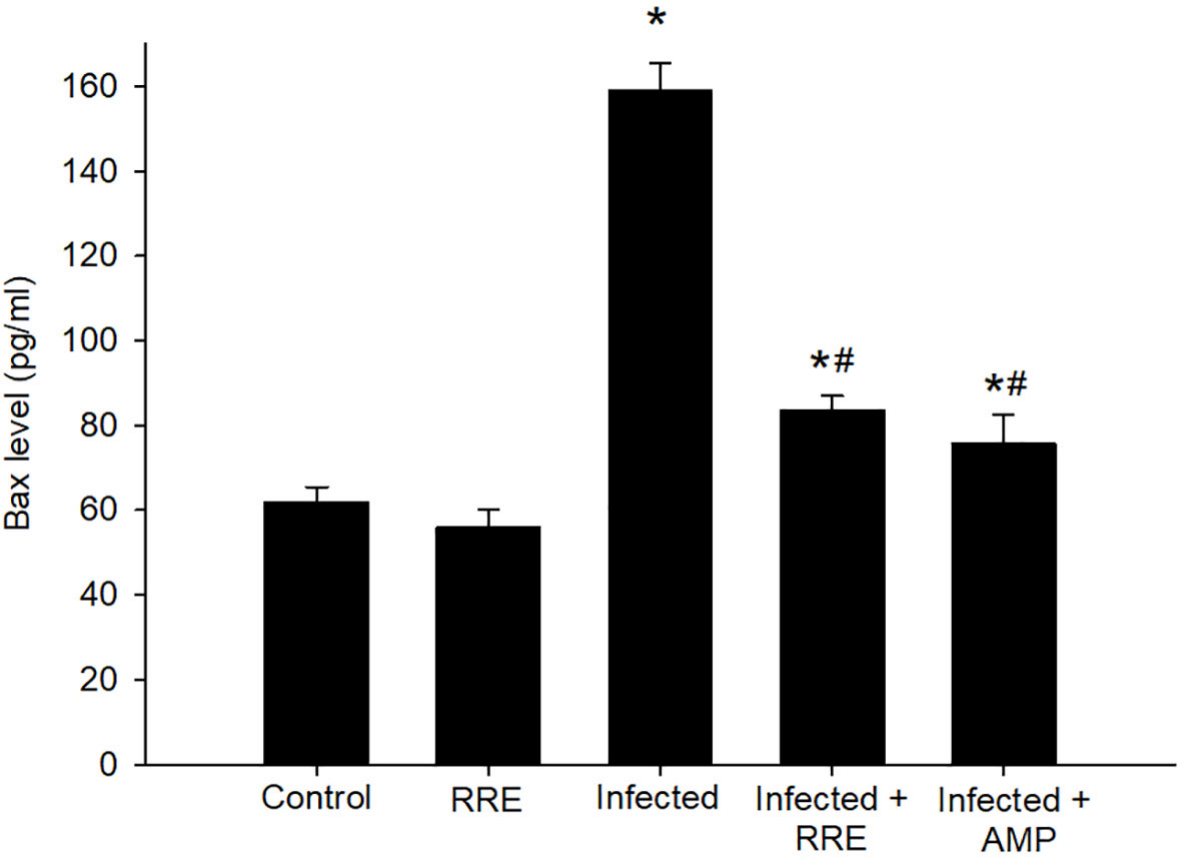
Bax level in the jejunal samples from the different experimental mice groups. ^*^ significant change (p ≤ 0.05) concerning the control group, ^#^ significance change (p ≤ 0.05) concerning the infected group with *E. papillata*.

**Figure 11:**
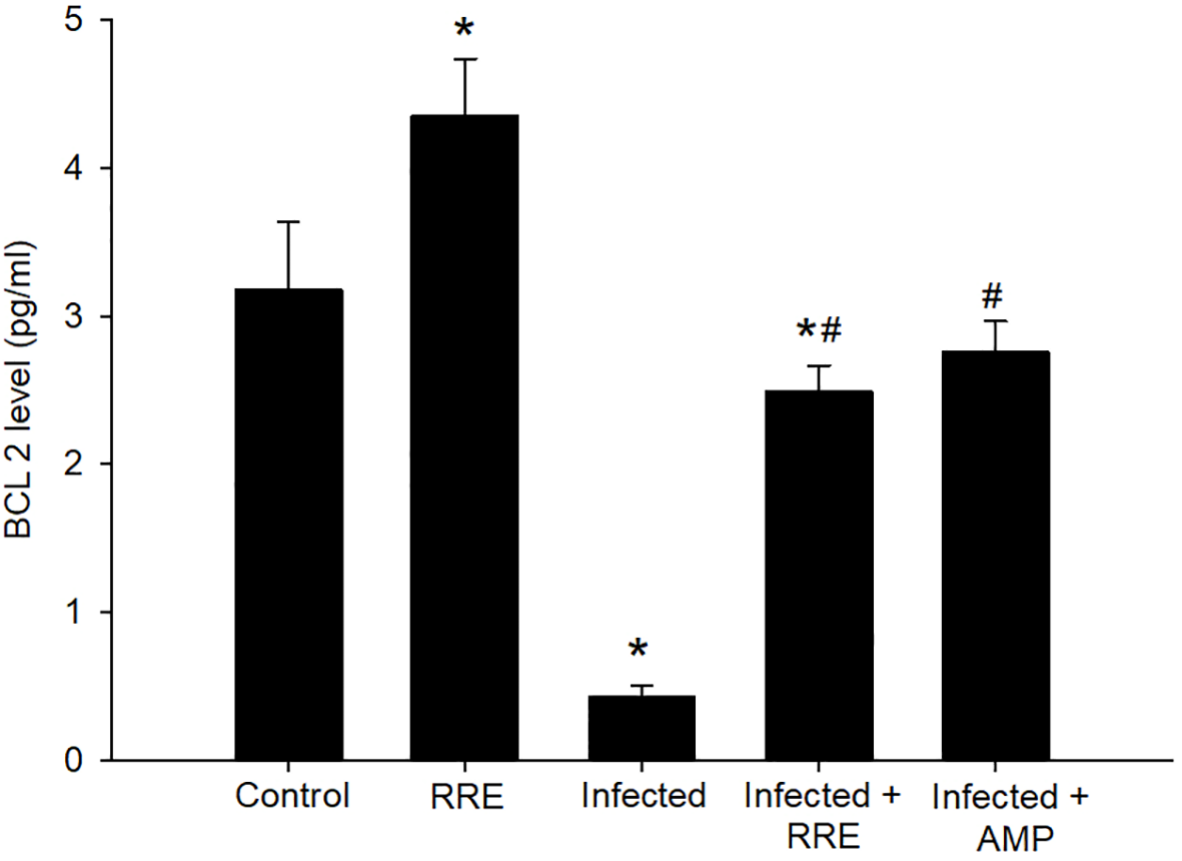
BCL2 level in the jejunal samples from the different experimental mice groups. ^*^ significant change (p ≤ 0.05) concerning the control group, ^#^ significance change (p ≤ 0.05) concerning the infected group with *E. papillata*.

Our findings further investigated the role of RRE in infection-induced apoptosis, by evaluating the level of Bax using ELISA (**Figure 10**). *Eimeria* infection induced a highly significant increase in the Bax level (159.05 ± 6.50 pg/ml) in comparison to the control group. RRE treatment, however, significantly lowered the *E. papillata*-induced increase in Bax level (83.68 ± 3.24 pg/ml) compared to the infected group. While BCL2 was found to be significantly decreased after *Eimeria* infection (0.42 ± 0.07 pg/ml) but significantly increased again upon treatment with RRE (2.49 ± 0.17 pg/ml) (**Figure 11**).

**Figure 12:**
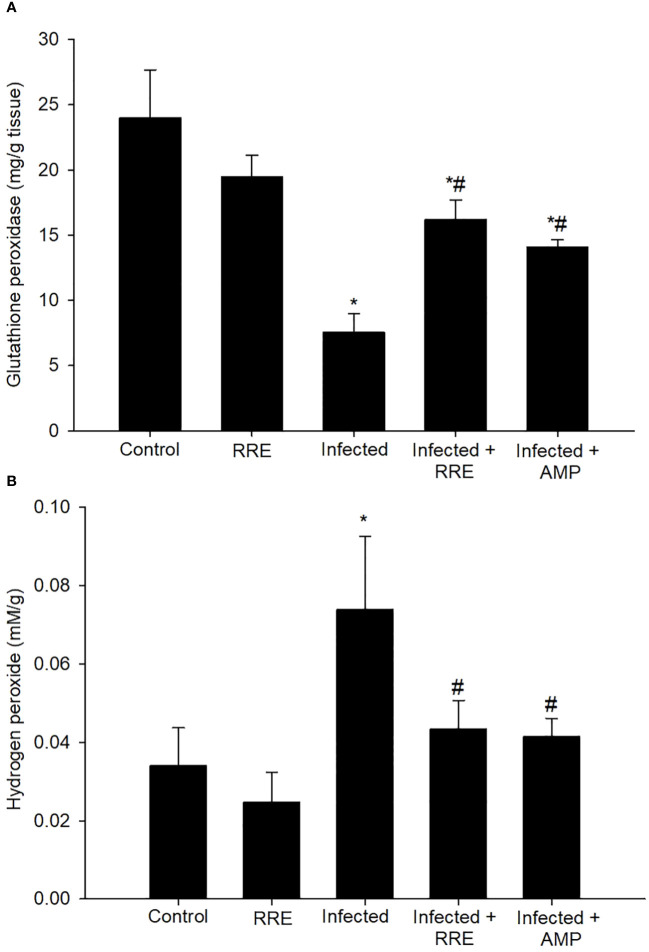
Levels of **(A)** glutathione peroxidase and **(B)** hydrogen peroxide in the different experimental mice groups. ^*^ significant change (p ≤ 0.05) concerning the control group, ^#^ significance change (p ≤ 0.05) concerning the infected group with *E*. *papillata*.

The *Eimeria* infection caused an unbalance in the oxidative status in the jejunum of mice. This was demonstrated by the estimation of GPx and H_2_O_2_ levels (**Figure 12**). There was a significant reduction in GPx from 23.99 ± 3.68 mg/g tissue in the control group to 7.53 ± 1.45 mg/g tissue in the infected group. However, infection with *E. papillata* induced cellular damage with a significant elevation in the reactive oxygen species concentration of H_2_O_2_ (0.07 ± 0.01 mM/g). RRE caused clear amelioration in the level of GPx (16.21 ± 1.48 mg/g tissue) and H_2_O_2_ (0.043 ± 0.007 mM/g) in the jejunal tissue compared to the infected group.

## Discussion

Coccidiosis is caused by *Eimeria* protozoan parasites and affects wild and domestic animals. Antieimerial agents from plant sources are currently used to control coccidiosis because of drug resistance (33). In this study, the pharmacological activities of rhatany root extract were investigated against *E. papillata* via *in vitro* and *in vivo* examinations. RRE possessed many active phytochemical compounds by GC-MS analysis such as oleic acid, β- and γ- sitosterol, which are well known for their medicinal activities such as anti-inflammatory (34, 35), anti-apoptotic (36, 37), antiparasitic (38), and antioxidant (35, 39) activities.

This study showed that RRE has a dose-dependent *in vitro* anti-eimerial effect on the *Eimeria* oocyst sporulation. RRE (200 mg/ml) has more than 50% significant values for inhibiting oocyst sporulation at different intervals, in comparison to other inhibitors. These results might be due to active compounds of RRE which impaired the oocyst wall functions leading to leakage of cellular constituents, which agreed with data obtained by Alamari et al. (21). Similarly, de Oliveira et al. (38) mentioned that oleic acid interacts with parasitic protozoa lipid membranes and deranges the electron transport chain and oxidative phosphorylation, as a result, it could be used as a complementary therapy for the treatment of various infectious diseases. During experimental *Eimeria* infection, the shedding rate of oocysts reached its peak at the 5^th^ day p.i. Upon treatment, RRE plays a vital role in the reduction of the oocysts number in mice faeces by containing some active ingredients that disrupt the cell membrane of parasitic *Eimeria* stages leading to impairing the ability to invade, replication, and development within the mice jejunal tissue. Abdel-Gaber et al. (14) and Alamari et al. (21) reported the antieimerial efficacy of rhatany root extract against *E. papillata-*induced experimental infection in mice. Moreover, the reduction in the oocyst output in the mice faeces, after treatment with roots obtained from natural plant sources, has been investigated in a previous study by Thagfan et al. (6) using *Salvadora persica* roots extract as an anti-eimerial agent.

One of the cellular immune responses in the intestine is the goblet cell response (40). During the *Eimeria* infection, the reduction in the number of goblet cells was observed herein, which agreed with Yunus et al. (41) reported that this reduction might be due to parasite damage to the stem cell population at the base of the intestinal crypts. Cheng and Leblond (42) stated that muco-secretory cells (goblet cells) arise by mitosis from pluripotent stem cells at the jejunal crypt. Linh et al. (43), reported that these stem cells, during the *Eimeria* infection, are parasitized and become unable to produce goblet cells, which could explain the significant reduction in their numbers in the infected group. Upon RRE treatment, the number of goblet cells was elevated which affected the infected mice’s susceptibility to opportunistic *Eimeria*’s ability to interact with or penetrate the target epithelium, resulting in depletion in the number of oocysts in mice feces. The possible hypothesis for the restoration of the number of goblet cells, after *Eimeria* infection, is the presence of active phytochemical compounds in RRE. This in line with Carrillo et al. (44) confirmed that oleic acid represents a major supplier for the full reconversion of cancerous/infected cells into healthy intestinal cells in restoration of goblet cells. Improvements in the induced hypoplasia of goblet cells due to infection with *Eimeria* parasite have been investigated using several medicinal plants such as *Azadirachta indica* (27), *Ziziphus spina*-*christi* (8), *Salvadora persica* (6), *Astragalus membranaceus* (11), and *Zingiber officinale* (7).

Our findings indicated that the expression of the goblet cell MUC2 gene was downregulated during *Eimeria* infection which is widely expressed in the goblet cells of the small intestine, which agreed with Forder et al. (45), Kim and Khan (46), and Yang and Yu (47). Larsson et al. (48) and Boltin et al. (49) stated that the MUC2 gene is the first line of innate host defense and is responsible for the regulation of mucin secretion and immune/inflammatory response against pathogen-induced injury. Herein, it has shown that RRE treatment interferes with the *Eimeria* parasite development in the jejunum and consequently upregulates both the number of goblet cells and the expression of its specific gene (MUC2) which helps to improve the inflammatory response to infectious diseases, which is consistent with Larsson et al. (48), Gum et al. (50), and Dkhil et al. (27).

Moreover, there was a significant elevation of MPO level which is a marker for a neutrophil’s alteration due to infection with the highly pathogenic *E. papillata*. This may relate to the critical role of neutrophils in host immunity during intestinal epithelium invasion by the developmental *Eimeria* stages. After treatment, the RRE attenuates the inflammatory response, since it suppresses the release of the infection-induced MPO level nearly to the normal status of the control group. Al-Quraishy et al. (51), Amer et al. (52), and Abbas et al. (53) reported that medicinal plants rich in antioxidant compounds have also shown remarkable immunomodulatory effects. This is in line with Yu et al. (54) reported that oleic acid shows anti-inflammatory activity toward activated neutrophils which confirms the role of RRE during eimerian infections. Similarly, the results of Naikwadi et al. (34) and Zhang et al. (35) demonstrate the antioxidative and anti-inflammatory activities of β- and γ- sitosterol and suggest that it may be useful for the treatment of various inflammatory diseases.

Lüder et al. (55) and Balamurugan et al. (56) reported that the host’s response against intracellular parasitic diseases may be controlled via different apoptotic pathways which aid in the removal of infected cells. Moreover, Liu et al. (57) reported that the phases of gamogony and sporogony induced pro-apoptotic markers and downregulated the anti-apoptotic marker. In this study, the pro-apoptotic markers caspase-3 and Bax significantly elevated, indicating the death of jejunal cells after *Eimeria* infection. These results agreed with previous studies by Dkhil et al. (27), Metwaly et al. (58), and Alkhudhayri et al. (59) stated that apoptotic cells in the mice jejuna were elevated after infection with *E. papillata*. Rossé et al. (60) reported that Bax is responsible for inducing cytochrome c release and caspase activation, which in turn, leads to cell death. Treatment with RRE induced apoptotic alterations in jejunal cells by the reduction of the pro-apoptotic markers which states the anti-apoptotic activity of this extract, this data is similar to that reported by Abdel-Gaber et al. (12) when using biosynthesized selenium nanoparticles using *Azadirachta indica* leaves extract as an anti-eimerial and anti-apoptotic agent after *E. papillata* infection.

Furthermore, Del Cacho et al. (61) reported that the *Eimeria* parasite could protect infected host cells from apoptosis by promoting the expression of the anti-apoptotic protein BCL2 during the process of the maturation of schizonts at the initial stage of the infection, while Jiao et al. (62) stated that, at late stages of infection, BCL2 expression was significantly reduced to enable the escape of the merozoites. This study revealed that on day 5 p.i., *Eimeria* infection suppressed the expression of BCL2. Similarly, Abdel-Tawab et al. (11) reported that the expression of BCL2 protein, in *E. papillata* parasitized tissue, was significantly reduced on day 5 after infection. Rasul et al. (63) mentioned that the reduction in the expression of anti-apoptotic proteins BCL-2 and BCL-X_L_ accelerates host-cell apoptosis. In our study, oral treatment with RRE significantly increased the level of BCL2. This may be due to the role of oleic acid and β-Sitosterol that restores the cell from apoptosis toward survival by attenuating the disturbance in the balance of anti-apoptotic (BCL-2) and pro-apoptotic (Bax) as well as the activation of caspase-3, as stated with Ahn et al. (36) and Vundru et al. (37).

In this study, the oxidant/antioxidant status was also investigated during murine coccidiosis. In the present study, the infection with *E. papillata* induces oxidative stress by decreasing the activity of GPx, while increasing the level of H_2_O_2_. Similarly, Abdel-Tawab et al. (11), Abdel-Gaber et al. (12), Esch and Petersen (64), Masood et al. (65), Dkhil et al. (66), and El-Ghareeb et al. (67) clarified that the antioxidant defense system’s imbalance due to *Eimeria* infection contributes to adverse cellular effects. Administration of RRE ameliorates the oxidative injury caused by the *Eimeria* parasite in the mice jejunum by normalizing the values of GPx and H_2_O_2_. This is in line with previous research that has ascribed the pronounced potential effects of RRE to the antioxidant activity of the biologically active ingredients, with special reference to oleic acid and β-Sitosterol, of this root extract, which might have beneficial effects in the treatment of various infectious diseases (14, 16, 18, 21, 35, 39).

## Conclusion

Collectively, rhatany root extract exhibits significant anti-eimerial, antioxidant, anti-apoptotic, and anti-inflammatory activities serving to protect the host tissue from injuries induced by *E. papillata* and it is, therefore, highly recommended for use as an alternative to the anti-eimerial drugs against murine coccidiosis. The limitations of our study lie in the scope of mechanistic elucidation. While we have shown the significant impact of RRE on regulating apoptosis in *Eimeria papillata* host cells, our investigation offers preliminary insight. We acknowledge that our study does not comprehensively dissect the underlying mechanisms through which RRE exerts its effects. Further research endeavors are warranted to delve deeper into these mechanisms, facilitating a more thorough understanding of the therapeutic potential of RRE in combating coccidiosis. Such studies would contribute to a broader comprehension and validation of RRE’s efficacy and safety profile, thereby enhancing its feasibility as a natural product for coccidiosis control measures.

## Data availability statement

The original contributions presented in the study are publicly available. This data can be found here: https://figshare.com/articles/figure/DATA_MS/25903219.

## Ethics statement

This study was supported by the Researchers Supporting Project (RSP2024R25), King Saud University, Riyadh, Saudi Arabia. The studies were conducted in accordance with the local legislation and institutional requirements.

## Author contributions

SA: Investigation, Methodology, Writing – original draft, Writing – review & editing. RA: Conceptualization, Formal analysis, Funding acquisition, Investigation, Methodology, Validation, Writing – original draft, Writing – review & editing. GA: Formal analysis, Methodology, Writing – original draft. AM: Investigation, Writing – original draft, Writing – review & editing. SE: Data curation, Writing – original draft, Writing – review & editing. EA: Investigation, Methodology, Writing – original draft. MD: Formal analysis, Investigation, Methodology, Writing – original draft, Writing – review & editing.

## References

[1] Alajmi F Al-Otaibi T Al-Quraishy S Al-Shaebi EM Al-Hoshani N Dkhil MA. *Persea americana* extract protects intestinal tissue from *Eimeria papillata*-induced murine infection. BMC Veterinary Res. (2023) 19:248. doi: 10.1186/s12917-023-03810-1 PMC10683183 38017513

[2] Dkhil MA Abdel-Maksoud MA Al-Quraishy S Abdel-Baki AS Wunderlich F. Gene expression in rabbit appendices infected with *Eimeria coecicola* . Veterinary Parasitol. (2012) 186:222–8. doi: 10.1016/j.vetpar.2011.11.031 22154972

[3] Al-Quraishy S Dkhil MA Alkhudhayri AA. Effects of the electromagnetic radiation on oocysts of *Eimeria papillata* infecting mice. Afr J Microbiol Res. (2011) 5:2755–9. doi: 10.5897/AJMR

[4] Kim YS Ho SB. Intestinal goblet cells and mucins in health and disease: recent insights and progress. Curr Gastroenterol Rep. (2010) 12:319–30. doi: 10.1007/s11894-010-0131-2 PMC2933006 20703838

[5] Noack S Chapman HD Selzer PM. Anticoccidial drugs of the livestock industry. Parasitol Res. (2019) 118:2009–26. doi: 10.1007/s00436-019-06343-5 PMC6611755 31152233

[6] Thagfan FA Dkhil MA Al-Quraishy S. *In vivo* anticoccidial activity of *Salvadora persica* root extracts. Pakistan J Zoology. (2017) 49:53–7. doi: 10.17582/journal.pjz/2017.49.1.51.55 PMC6733712 31516352

[7] Mubaraki MA Thagfan FA Alkhudhayri A Al-Shaebi EM Maodaa SN Abdel-Gaber R. *Zingiber officinale* supplementation suppresses eimeriosis and regulates goblet cell response. Saudi J Biol Sci. (2022) 29:3403–7. doi: 10.1016/j.sjbs.2022.02.025 PMC9280212 35844435

[8] Alzahrani F Al-Shaebi EM Dkhil MA Al-Quraishy S. *In vivo* anti-*Eimeria* and in *vitro* anthelmintic activity of *Ziziphus spina*-*christi* leaf extracts. Pakistan J Zoology. (2016) 48:409–13.

[9] Hussain K Iqbal Z Abbas RZ Khan MK Saleem MK. Immunomodulatory activity of *Glycyrrhiza glabra* extract against mixed *Eimeria* infection in chickens. Int J Agric Biol. (2017) 19:928–32. doi: 10.17957/IJAB

[10] Al-Quraishy S Thagfan FA Al-Shaebi EM Qasem M Abdel-Gaber R Dkhil MA. *Salvadora persica* protects mouse intestine from eimeriosis. Braz J Vet Parasitol Jaboticabal. (2019) 28:605–12. doi: 10.1590/s1984-29612019068 31721926

[11] Abdel-Tawab H Abdel-Haleem HM Abdel-Baki AAS Al-Quraishy S El-Mallah AM. Anticoccidial and antioxidant activities of *Moringa oleifera* leaf extract on murine intestinal eimeriosis. Acta Parasitologica. (2020) 65:823–30. doi: 10.2478/s11686-020-00219-w 32472400

[12] Abdel-Gaber R Hawsah MA Al-Otaibi T Alojayri G Al-Shaebi EM Mohammed OB. Biosynthesized selenium nanoparticles to rescue coccidiosis mediated oxidative stress, apoptosis and inflammation in the jejunum of mice. Front Immunol. (2023) 14:1139899. doi: 10.3389/fimmu.2023.1139899 36875142 PMC9982015

[13] Christenhusz MJM Byng JW. The number of known plants species in the world and its annual increase. Phytotaxa. (2016) 261:201–17. doi: 10.11646/phytotaxa.261.3

[14] Abdel-Gaber R Alamari G Dkhil MA Meryk A Al-Shaebi EM Al-Quraishy S. *Krameria lappacea* root extract’s anticoccidial properties and coordinated control of CD4 T cells for IL-10 production and antioxidant monitoring. Front Immunol. (2024) 15:1404297. doi: 10.3389/fimmu.2024.1404297 38751432 PMC11094240

[15] Carini M Aldini G Orioli M Facino RM. Antioxidant and photoprotective activity of a lipophilic extract containing neolignans from *Krameria triandra* roots. Planta Med. (2002) 68:193–7. doi: 10.1055/s-2002-23167 11914952

[16] Baumgartner L Schwaiger S Stuppner H. Quantitative analysis of anti-inflammatory lignan derivatives in *Ratanhiae radix* and its tincture by HPLC-PDA and HPLC-MS. J Pharm Biomed Anal. (2011) 56:546–52. doi: 10.1016/j.jpba.2011.06.016 PMC3157606 21783335

[17] Heiss EH Baumgartner L Schwaiger S Heredia RJ Atanasov AG Rollinger JM. Ratanhiaphenol III from Ratanhiae radix is a PTP1B inhibitor. Planta Med. (2012) 78:678–81. doi: 10.1055/s-0031-1298242 PMC3523387 22307937

[18] Al-Oqail MM. Anticancer efficacies of *Krameria lappacea* extracts against human breast cancer cell line (MCF-7): Role of oxidative stress and ROS generation. Saudi Pharm J. (2021) 29:244–51. doi: 10.1016/j.jsps.2021.01.008 PMC8084728 33981173

[19] Zabka M. Antifungal efficacy and convenience of *Krameria lappacea* for the development of botanical fungicides and new alternatives of antifungal treatment. Agronomy. (2022) 12:2599. doi: 10.3390/agronomy12112599

[20] Velasco-Lezama R Aguilar-Carrillo MF Tapia-Aguilar R Velázquez-Vázquez MDL Cerón-Ramírez R Santana-Carrillo J. Determination of the antibacterial activity of *Krameria pauciflora* (Rose). J Drug Delivery Ther. (2023) 13:105–9. doi: 10.22270/jddt.v13i3.5996

[21] Alamari G Abdel-Gaber R Al-Shaebi E Al-Quraishy E. Anticoccidial and jejunum-protective effects of *Krameria lappacea* roots extract on experimental *Eimeria papillata* infection. Microscopy Res Technique. (2024) 87(7):1467–1478. doi: 10.1002/jemt.24531 38407507

[22] Kanthal LK Dey A Satyavathi K Bhojaraju P. GC-MS analysis of bio-active compounds in methanolic extract of *Lactuca runcinata* DC. Pharmacognosy Res. (2014) 6:58–61. doi: 10.4103/0974-8490.122919 24497744 PMC3897010

[23] Thagfan FA Al-Megrin WA Al-Shaebi EM Al-Quraishy S Dkhil MA. Protective role of *Morus nigra* leaf extracts against murine infection with *Eimeria papillata* . Combinatorial Chem High Throughput Screening. (2020) 24:1603–8. doi: 10.2174/1386207323666200903152811 32885749

[24] Villareal-García LE Oranday-Cárdenas A de la Garza-Ramos MA Rivas-Morales C Verde-Star MJ Gómez-Treviño JA. Neolignanos de *Krameria ramosissima* (A. Gray) S. Watson con actividad contra *Porphyromonas gingivalis*, evaluación citotóxica y mutagénica. Rev Mex Cienc Farm. (2014) 45:69–76.

[25] Abakar AD Seri HI Ismail AA Musa HH. Comparative efficacy of selected anticoccidial drugs in ambarorow sheep naturally infected with enteric coccidia in South Darfur, Sudan. Sudan J Vet Res. (2005) 20:61–7.

[26] Adam H Caihak G. Grosses zoologisches parktikum tell. In: Arbeitsmethoden der makroskopischen und mikroskopischen anatomic Mit 283 Abbildungen Gustav. Fischer Verlag Stuttgart (1964).

[27] Dkhil MA Al-Quraishy S Abdel Moneim AE Delic D. Protective effect of *Azadirachta indica* extract against *Eimeria papillata*-induced coccidiosis. Parasitol Res. (2013) 112:101–6. doi: 10.1007/s00436-012-3109-1 22972359

[28] Dkhil MA Abdel Moneim AE Bauomy AA Khalil M Al-Shaebi EM Al-Quraishy S. Chlorogenic acid prevents hepatotoxicity in arsenic-treated mice: role of oxidative stress and apoptosis. Mol Biol Rep. (2020) 47:1161–71. doi: 10.1007/s11033-019-05217-4 31820315

[29] Paglia DE Valentine WN. Studies on the quantitative and qualitative characterization of erythrocyte glutathione peroxidase. J Lab Clin Med. (1967) 70:158–69. doi: 10.5555/uri:pii:0022214367900765 6066618

[30] Aebi HU. Methods in Enzymatic Analysis. New York: Academic press (1984) p. 121–6.

[31] Bradley PP Priebat DA Christensen RD Rothstein G. Measurement of cutaneous inflammation: estimation of neutrophil content with an enzyme marker. J Invest Dermatol. (1982) 78:206–9. doi: 10.1111/1523-1747.ep12506462 6276474

[32] Livak KJ Schmittgen TD. Analysis of relative gene expression data using real-time quantitative PCR and the 2(-Delta C(T)) Method. Methods. (2001) 25:402–8. doi: 10.1006/meth.2001.1262 11846609

[33] Muthamilselvan T Kuo TF Wu YC Yang WC. Herbal remedies for coccidiosis control: a review of plants, compounds, and anticoccidial actions. Evidence-Based Complement Altern Med. (2016) 2016:1–19. doi: 10.1155/2016/2657981 PMC4939967 27429634

[34] Naikwadi P Phatangare ND Mane DV. Active anti-inflammatory potency of γ-sitosterol from *woodfordia floribunda salisb* . J Plant Sci Res. (2022) 38:691–700.

[35] Zhang P Liu N Xue M Zhang M Liu W Xu C. Anti-inflammatory and antioxidant properties of β-sitosterol in copper sulfate-induced inflammation in Zebrafish (*Danio rerio*). Antioxidants. (2023) 12:391. doi: 10.3390/antiox12020391 36829951 PMC9952786

[36] Ahn JH Kim MH Kwon HJ Choi SY Kwon HY. Protective effects of oleic acid against palmitic acid-induced apoptosis in pancreatic AR42J cells and its mechanisms. Korean J Physiol Pharmacol. (2013) 17:43–50. doi: 10.4196/kjpp.2013.17.1.43 23440052 PMC3579104

[37] Vundru SS Kale RK Singh RP. β-sitosterol induces G1 arrest and causes depolarization of mitochondrial membrane potential in breast carcinoma MDA-MB-231 cells. BMC Complement Altern Med. (2013) 13:280. doi: 10.1186/1472-6882-13-280 24160369 PMC3819702

[38] de Oliveira RN Campos PM Pinto RMC Mioduski J Santos RD Hustus B. The promising antischistosomal activity of oleic acid-loaded polymeric nanocapsules for oral administration. J Drug Deliv Sci Technol. (2021) 63:102429. doi: 10.1016/j.jddst.2021.102429

[39] Wei CC Yen PL Chang ST Cheng PL Lo YC Liao VHC. Antioxidative activities of both oleic acid and *Camellia tenuifolia* seed oil are regulated by the transcription factor DAF-16/FOXO in *Caenorhabditis elegans* . PloS One. (2016) 11:e0157195. doi: 10.1371/journal.pone.0157195 27275864 PMC4898728

[40] Dkhil MA Thagfan FA Morad MY Al-Shaebi EM Elshanat S Bauomy AA. Biosynthesized silver nanoparticles have anticoccidial and jejunum-protective effects in mice infected with *Eimeria papillata* . Environ Sci Pollut Res. (2023) 30:44566–77. doi: 10.1007/s11356-023-25383-0 PMC9873539 36694067

[41] Yunus M Horii Y Makimura S Smith AL. Murine goblet cell hypoplasia during *Eimeria pragensis* infection is ameliorated by clindamycin treatment. J Vet Med Sci. (2005) 67:311–5. doi: 10.1292/jvms.67.311 15805736

[42] Cheng H Leblond CP. Origin, differentiation and renewal of the four main epithelial cell types in the mouse small intestine. II. Mucous cells. Am J Anat. (1974) 141:481–501. doi: 10.1002/aja.1001410404 4440633

[43] Linh BK Hayashi T Horii Y. *Eimeria vermiformis* infection reduces goblet cells by multiplication in the crypt cells of the small intestine of C57BL/6 mice. Parasitol Res. (2009) 104:789–94. doi: 10.1007/s00436-008-1256-1 19005680

[44] Carrillo C Del Ma Cavia M Alonso-Torre S. Role of oleic acid in immune system; mechanism of action; a review. Nutr Hosp. (2012) 27:978–90. doi: 10.3305/nh.2012.27.4.5783 23165533

[45] Forder R Nattrass G Geier M Hughes R Hynd P. Quantitative analyses of genes associated with mucin synthesis of broiler chickens with induced necrotic enteritis. Poult Sci. (2012) 91:1335–41. doi: 10.3382/ps.2011-02062 22582290

[46] Kim JJ Khan WI. Goblet cells and mucins: role in innate defense in enteric infections. Pathogens. (2013) 2:55–70. doi: 10.3390/pathogens2010055 25436881 PMC4235714

[47] Yang S Yu M. Role of goblet cells in intestinal barrier and mucosal immunity. J Inflammation Res. (2021) 14:3171–83. doi: 10.2147/JIR.S318327 PMC8286120 34285541

[48] Larsson JM Karlsson H Crespo JG Johansson ME Eklund L Sjovall H. Altered O-glycosylation profile of MUC2 mucin occurs in active ulcerative colitis and is associated with increased inflammation. Inflamm Bowel Dis. (2011) 17:2299–307. doi: 10.1002/ibd.21625 21290483

[49] Boltin D Perets TT Vilkin A Niv Y. Mucin function in inflammatory bowel disease, an update. J Clin Gastroenterol. (2013) 47:106–11. doi: 10.1097/MCG.0b013e3182688e73 23164684

[50] Gum JR Hicks JW Gillespie AM Carlson EJ Kömüves L Karnik S. Goblet cell-specific expression mediated by the MUC2 mucin gene promoter in the intestine of transgenic mice. Am J Physiol. (1999) 276:G666–76. doi: 10.1152/ajpgi.1999.276.3.G666 10070043

[51] Al-Quraishy S Metwaly MS Dkhil MA Abdel-Baki S Wunderlich F. Liver response of rabbits to *Eimeria coecicola* infections. Parasitol Res. (2012) 110:901–11. doi: 10.1007/s00436-011-2574-2 21822680

[52] Amer OS Dkhil MA Hikal WM Al-Quraishy S. Antioxidant and anti-inflammatory activities of pomegranate (*Punica granatum*) on *Eimeria papillata*-induced infection in mice. BioMed Res Int. (2015) 2015:219670. doi: 10.1155/2015/219670 25654088 PMC4310320

[53] Abbas A Iqbal Z Abbas RZ Khan MK Khan JA Sindhu ZUD. *In vivo* anticoccidial effects of Beta vulgaris (sugar beet) in broiler chickens. Microb Pathog. (2017) 111:139–44. doi: 10.1016/j.micpath.2017.07.052 28826766

[54] Yu HP Liu FC Umoro A Lin ZC Elzoghby AO Hwang TL. Oleic acid-based nanosystems for mitigating acute respiratory distress syndrome in mice through neutrophil suppression: how the particulate size affects therapeutic efficiency. J Nanobiotechnology. (2020) 18:25. doi: 10.1186/s12951-020-0583-y 32005196 PMC6995149

[55] Lüder CG Gross U Lopes MF. Intracellular protozoan parasites and apoptosis: Diverse strategies to modulate parasite-host interactions. Trends Parasitol. (2001) 17:480–6. doi: 10.1016/s1471-4922(01)02016-5 11587962

[56] Balamurugan K Rajaram R Ramasami T Narayanan S. Chromium (III)-induced apoptosis of lymphocytes: Death decision by ROS and src-family tyrosine kinases. Free Radic Biol Med. (2002) 33:1622–40. doi: 10.1016/S0891-5849(02)01115-2 12488131

[57] Liu J Deng M Lancto CA Abrahamsen MS Ruther-Ford MS Enomoto S. Biphasic modulation of apoptotic pathways in *Cryptosporidium parvum* infected human intestinal epithelial cells. Infect Immun. (2009) 77:837–49. doi: 10.1128/IAI.00955-08 PMC2632021 19075026

[58] Metwaly MS Dkhil MA Al-Quraishy S. Anti-coccidial and anti-apoptotic activities of palm pollen grains on *Eimeria papillata*-induced infection in mice. Biologia. (2014) 69:254–9. doi: 10.2478/s11756-013-0297-9

[59] Alkhudhayri A Al-Shaebi EM Qasem MAA Murshed M Mares MM Al-Quraishy S. Antioxidant and anti-apoptotic effects of selenium nanoparticles against murine eimeriosis. Acad Bras Cienc. (2020) 92:e20191107. doi: 10.1590/0001-3765202020191107 32520220

[60] Rossé T Olivier R Monney L Rager M Conus S Fellay I. Bcl-2 prolongs cell survival after Bax-induced release of cytochrome c. Nature. (1998) 391:496–9. doi: 10.1038/35160 9461218

[61] Del Cacho E Gallego M Lopez-Bernad F Quílez J Sánchez-Acedo C. Expression of anti-apoptotic factors in cells parasitized by second-generation schizonts of *Eimeria tenella* and *Eimeria necatrix* . Vet Parasitol. (2004) 125:287–300. doi: 10.1016/j.vetpar.2004.07.017 15482885

[62] Jiao J Yang Y Liu M Li J Cui Y Yin S. Artemisinin and *Artemisia annua* leaves alleviate *Eimeria tenella* infection by facilitating apoptosis of host cells and suppressing inflammatory response. Vet Parasitol. (2018) 254:172–7. doi: 10.1016/j.vetpar.2018.03.017 29657004

[63] Rasul A Ding C Li X Khan M Yi F Ali M. Dracorhodin perchlorate inhibits P13K/Akt and NF-kB activation, up-regulates the expression of p53, and enhances apoptosis. Apoptosis. (2012) 17:1104–19. doi: 10.1007/s10495-012-0742-1 22711363

[64] Esch KJ Petersen CA. Transmission and epidemiology of zoonotic protozoal diseases of companion animals. Clin Microbiol Rev. (2013) 26:58–85. doi: 10.1128/CMR.00067-12 23297259 PMC3553666

[65] Masood S Abbas RZ Iqbal Z Mansoor MK Sindghu Z Zia MA. Role of natural antioxidants for the control of coccidiosis in Poultry. Pak Vet J. (2013) 33:401–7.

[66] Dkhil MA Metwaly MS Al-Quraishy S Sherif NE Delic D Al Omar SY. Anti-*Eimeria* activity of berberine and identification of associated gene expression changes in the mouse jejunum infected with *Eimeria papillata* . Parasitol Res. (2015) 114:1581–93. doi: 10.1007/s00436-015-4344-z 25663104

[67] El-Ghareeb WR Kishawy ATY Anter RGA Aboelabbas Gouda A Abdelaziz WS Alhawas B. Novel antioxidant insights of myricetin on the performance of broiler chickens and alleviating experimental infection with Eimeria spp.: crosstalk between oxidative stress and inflammation. Antioxidants. (2023) 12:1026. doi: 10.3390/antiox12051026 37237892 PMC10215077

