# Supplementary material for: Fighting eimeriosis by using the anti-eimerial and anti-apoptotic properties of rhatany root extract
Source: Front Immunol. 2024 Jul 11;15:1430960. doi: 10.3389/fimmu.2024.1430960 (PMC11269128; doi:10.3389/fimmu.2024.1430960)
Supplement: Supplementary file 1 [file DataSheet_1.docx]

**Figure S1.** GC-MS Chromatogram of aqueous RRE
